# Salicylic Acid and Melatonin Synergy Enhances Boron Toxicity Tolerance via AsA–GSH Cycle and Glyoxalase System Regulation in Fragrant Rice

**DOI:** 10.3390/metabo14100520

**Published:** 2024-09-26

**Authors:** Muhammad Imran, Emilie Widemann, Sarfraz Shafiq, Ali Bakhsh, Xiaoyuan Chen, Xiangru Tang

**Affiliations:** 1 State Key Laboratory for Conservation and Utilization of Subtropical Agro-Bioresources, College of Agriculture, South China Agricultural University, Guangzhou 510642, China; muhammadimran@scau.edu.cn; 2 Guangdong Provincial Key Laboratory of Utilization and Conservation of Food and Medicinal Resources in Northern Region, Shaoguan University, Shaoguan 512005, China; chenxy88888@sgu.edu.cn; 3 Institut de Biologie Moléculaire des Plantes, CNRS-Université de Strasbourg, 67084 Strasbourg, France; ewidema4@uwo.ca; 4 Thompson Rivers University, Kamloops, BC V2C 0C8, Canada; sshafiq2@uwo.ca; 5 Department of Plant Breeding and Genetics, Ghazi University, Dera Ghazi Khan 32200, Pakistan; abakhsh@gudgk.edu.pk

**Keywords:** melatonin, salicylic acid, antioxidants, boron stress, fragrant rice, 2-acetyle-1-pyrroline

## Abstract

**Background:** Boron is an essential micronutrient for plant growth and productivity, yet excessive boron leads to toxicity, posing significant challenges for agriculture. Fragrant rice is popular among consumers, but the impact of boron toxicity on qualitative traits of fragrant rice, especially aroma, remains largely unexplored. The individual potentials of melatonin and salicylic acid in reducing boron toxicity are less known, while their synergistic effects and mechanisms in fragrant rice remain unclear. **Methods:** Thus, this study investigates the combined application of melatonin and salicylic acid on fragrant rice affected by boron toxicity. One-week-old seedlings were subjected to boron (0 and 800 µM) and then treated with melatonin and salicylic acid (0 and 100 µM, for 3 weeks). **Results:** Boron toxicity significantly impaired photosynthetic pigments, plant growth, and chloroplast integrity while increasing oxidative stress markers such as hydrogen peroxide, malondialdehyde, methylglyoxal, and betaine aldehyde dehydrogenase. Likewise, boron toxicity abridged the precursors involved in the 2-acetyl-1-pyrroline (2-AP) biosynthesis pathway. However, individual as well as combined application of melatonin and salicylic acid ameliorated boron toxicity by strengthening the antioxidant defense mechanisms—including the enzymes involved during the ascorbate–glutathione (AsA–GSH) cycle and glyoxalase system—and substantially improved 2-AP precursors including proline, P5C, Δ1-pyrroline, and GABA levels, thereby restoring the 2-AP content and aroma. These findings deduce that melatonin and salicylic acid synergistically alleviate boron toxicity-induced disruptions on the 2-AP biosynthesis pathway by improving the 2-AP precursors and enzymatic activities, as well as modulating the physio-biochemical processes and antioxidant defense system of fragrant rice plants. **Conclusions:** The findings of this study have the potential to enhance rice productivity and stress tolerance, offering solutions to improve food security and sustainability in agricultural practices, particularly in regions affected by environmental stressors.

## 1. Introduction

Boron is an essential micronutrient vital for plant growth, but its high solubility in soil and irrigation systems often leads to reduced productivity [1,2]. Consequently, boron fertilizers are commonly utilized in agricultural practices. However, maintaining a delicate balance between boron deficiency and excess is essential, as unregulated boron application can lead to toxicity, posing significant challenges compared to boron deficiency [3,4]. Moreover, in arid and semi-arid regions, boron accumulation in agricultural soils, exacerbated by water evaporation through capillary action, poses substantial risks to plant health despite its inherent abundance [5]. Factors contributing to rising boron levels include fertilizer usage, mining activities, and irrigation practices [6]. Boron toxicity disrupts photosynthesis and induces oxidative stress on lipids and proteins, impairing cellular functions and inhibiting nucleic acid and root cell division in plants [7,8]. Under stressful environments, plants accumulate ROS, which are encountered by antioxidant defense mechanisms [9,10]. Therefore, to stabilize ROS concentrations, plants have evolved various strategies via enzymatic and non-enzymatic defense systems [11]. One such compound, methylglyoxal (MG), synthesized by plants, is notorious for degrading lipids and proteins, thereby compromising cell membranes [12]. To counteract the adverse effects of methylglyoxal accumulation, plants initiate the glyoxalase system, which includes enzymes like glyoxalase I (Gly-I) and glyoxalase II (Gly-II), to eliminate methylglyoxal [13,14]. Research has demonstrated that bio-stimulants can alleviate the harmful impacts of various stressors, including boron stress, thereby enhancing plant resilience to adverse conditions [5,15]. Consequently, enhancing plant tolerance to boron-induced toxicity and devising phytoremediation techniques are increasingly recognized as beneficial strategies for cultivating plants in contaminated soils.

Phytohormones have emerged as powerful tools for counteracting stress in plants, functioning at low concentrations to orchestrate vital signals essential for plant resilience [16,17]. Notably, salicylic acid (SA) has garnered attention for its proven efficacy in mitigating oxidative stress [18]. Serving as a pivotal phytohormone and signaling molecule, SA plays a regulatory role in various physiological processes, including proline metabolism, photosynthesis, and the activation of antioxidant enzymes under stress conditions [19,20]. Numerous studies have documented SA’s ability to enhance the antioxidant defense system and alleviate oxidative stress in plants facing diverse stressors [21,22,23]. Through mechanisms such as the upregulation of SOD and the activation of H_2_O_2_ scavenging enzymes like peroxidase (POD), catalase (CAT), and ascorbate peroxidase (APX), SA facilitates cellular ROS detoxification [24]. Noteworthy is SA’s involvement in plant responses to a range of stressors, including salinity, lead, drought, boron toxicity, and cadmium stress [25,26,27,28,29]. However, despite its extensive role in stress mitigation, research examining SA-induced tolerance to boron toxicity in fragrant rice is lacking. On the other hand, melatonin, also known as N-acetyl-5-methoxytryptamine, serves as a vital animal hormone and indoleamine compound, regulating various physiological functions [30,31]. While melatonin has sparked significant interest in plant biology, its regulatory and functional roles remain partly understood. It has been linked to promoting plant growth and conferring protection against abiotic stresses across diverse crop species [30,32,33,34,35]. Acting as a broad-spectrum antioxidant, melatonin efficiently scavenges ROS and activates redox-sensitive regulatory pathways [36]. Its application has shown benefits in seedling germination, plant growth, and stress resistance in crops like watermelon, cucumber, kiwi, and Arabidopsis thaliana under various stressors such as salinity, drought, endoplasmic reticulum stress, and heat [32,37]. Melatonin’s interaction with ROS triggers the production of derivative compounds with potent antioxidant capacities, further enhancing its role in abiotic stress responses [32,36,38,39]. Ongoing discussions continue to explore melatonin’s dual function as both a growth regulator and an antioxidant [40,41]. Nevertheless, despite its extensive role in stress mitigation, research examining melatonin-induced tolerance to boron toxicity in fragrant rice is lacking.

Fragrant rice, renowned across Southeast Asia for its exquisite aroma and culinary allure [42,43], presents a compelling subject for investigating the repercussions of nutrient toxicity on plant characteristics [44]. Despite its widespread popularity among consumers, the impact of boron toxicity on the qualitative attributes of fragrant rice, particularly its aroma, remains largely unexplored. Boron toxicity effects and tolerance mechanisms have been investigated in various plant species [5,15], yet the synchronized activities of antioxidant defense and glyoxalase systems under boron toxicity have received limited attention. To test our hypothesis, we conducted a hydroponic study aimed at unraveling the underlying mechanisms behind the changes induced by boron toxicity in fragrant rice seedlings. Our objective was to shed light on the factors inhibiting the growth of fragrant rice seedlings under boron toxicity and to furnish practical insights for bolstering rice productivity. Through this investigation, we sought to provide valuable knowledge that could inform strategies for mitigating the detrimental effects of boron toxicity on fragrant rice and potentially enhance its cultivation and yield.

## 2. Material and Methods

### 2.1. Plant Material and Growth Environment

To ensure precise control over environmental variables and eliminate any potential soil-related influences on boron absorption in rice plants, we conducted a hydroponic experiment. Basmati rice seeds were surface sterilized with NaClO solution (5%) and rinsed with Milli-Q water. Afterward, the sterilized seeds were incubated in darkness at 30 °C on damp filter paper to facilitate germination. The seedlings with three emerged leaves were transferred to a growing environment comprising 80% R.H. and 16/8-h day/night photoperiod. The fragrant rice seedlings were transplanted to Yoshida nutrient solutions in plastic boxes with 1 L capacity [45]. Previously, it was reported that synergetic application of 100 μM of SA and MT can tolerate Cd toxicity by reducing the Cd uptake in safflower [46], while 50 μM of MT can alleviate the boron stress in rice [47]. Until now, the synergetic interaction of SA and MT against BT was limited in fragrant rice. Prior to the main experiment, a preliminary trial was conducted based on previous research to determine suitable concentrations of SA and MT for mitigating the uptake of boron and improving root length and aroma production. The trial tested 100 mgL^−1^ of SA and MT, which exhibited potential in alleviating BT stress. Based on these preliminary findings, five distinct treatments were established: CK (control), BT (800 μM), SA + BT (100 μM SA + 800 μM BT), MT + BT (100 μM MT + 800 μM BT), and SA + MT + BT (100 μM SA + 100 μM MT + 800 μM BT) under a completely randomized design with three replications. The nutrient solutions, including the treatment doses, were regularly renewed at three-day intervals until three-weeks-matured fragrant rice seedlings were harvested. At the end of this period, the chlorophyll content in the mature leaves was determined using a SPAD meter (SPAD-502, Minolta, Japan) [48], and the harvested rice seedlings were immediately frozen using liquid N_2_ and stored at −80 °C for subsequent morpho-physio-biochemical, and aromatic analysis. Morphological assessments were conducted with an Epson 12000XL scanner (Beijing, China) in transparent plastic trays (20 cm × 15 cm). Root and shoot lengths were measured and recorded individually. Root morphology analysis was performed using an Epson V700 root scanner integrated with WinRhizo software. The fresh weight of plants was determined by calculating the average weight of three randomly selected sample plants from each pot. For the dry weights of both the shoot and root, three pots were selected, and the plants were oven dried.

### 2.2. TEM and Confocal Microscopic Analysis

For TEM analysis, leaf segments (1–3 mm) were immersed for 6–8 h in 4% glutaraldehyde, post-fixed for 1 h in 1% OsO_4_, and rinsed with 0.2 mol/L PBS (pH 7.2) for 1–2 h. Dehydration was performed using a graded ethanol series (50% to 100%) and acetone. The dehydrated samples were filtered, embedded in Spurr’s resin, and the images were captured using TALOS L120C [49]. For LSCM analysis, fresh rice roots from various treatment groups were carefully collected and thoroughly cleaned using deionized water. Afterward, they were gently dried using tissue paper. The mature sections of the roots were specifically chosen and then embedded in a 4% agarose solution with a low melting point. To create semi-thin slices, the roots were cut to a thickness of 80 µm using a vibrating microtome (VT1200S, Leica, Nussloch, Germany). Subsequently, these slices were immersed in a solution of 10 µg/mL propidium iodide in H_2_O for a duration of 10 min, conducted in a dark environment. Following this staining, the slices were rinsed twice with H_2_O for 1 min each. The cross sections of the stained slices were then carefully positioned onto slides. A drop of PBS was added, and the samples were sealed using a cover glass. The resulting specimens were subjected to fluorescence observation utilizing a Leica SPE laser scanning confocal microscope. The images obtained were captured employing the Olympus FV10-ASW software (Ver.4.2b).

### 2.3. Determination of Hydrogen Peroxide and Malondialdehyde

To determine hydrogen peroxide (H_2_O_2_) levels, we followed the method of [50]. Fresh leaves (500 mg) were ground with 0.1% TCA and centrifuged at 12,000× *g* for 15 min. The absorbance was measured at 390 nm to determine H_2_O_2_ content. To assess lipid peroxidation, indicating malondialdehyde (MDA) levels, we employed the TBA reaction method outlined by Draper et al. [51].

### 2.4. Determination of Antioxidant Enzyme Activity

The fresh leaf samples (0.25 g) were homogenized with sodium phosphate buffer (0.05 M, pH 7.2). Then, the homogenate was separated by centrifuging at 12,000× *g* for 15 min. POD activity was assayed by following the method of [52], SOD activity was determined according to the method of [53], and CAT activity was assessed by measuring the initial rate of vanishing of H_2_O_2_ [54].

### 2.5. Determination of Glyoxalase System and Ascorbate-Glutathione Cycle

The glyoxalase I (Gly-I) and glyoxalase II (Gly-II) activities were determined according to the methods described by [55]. To assess APX activity, the plant extract (0.7 mL) was combined with EDTA (0.1 mM), 50 mM PBS buffer, and 0.5 mM of ascorbic acid, and the optical density change was monitored for 1 min at 290 nm [56]. To measure the monodehydroascorbate reductase (MDHAR) activity, the reaction solution included plant extract, 50 mM of Tris−HCl buffer, 0.5 Units of ascorbate oxidase, 0.2 mM NADPH, and 2.5 mM of AsA [57]. The activities of dehydroascorbate reductase (DHAR) and glutathione reductase (GR) were assessed according to the methods of [56] and [58], respectively.

### 2.6. Determination of the Enzymes of 2AP Biosynthesis

The activity of the enzymes involved during the 2AP biosynthesis in fragrant rice, i.e., PDH, P5CR, and BADH, was determined using the Plant ELISA kit (Mlbio, Shanghai, China). The determination steps were strictly followed as described in the instruction manual.

### 2.7. Determination of the 2AP Biosynthesis Precursors Content

The proline, 1-pyrroline, and methylglyoxal contents were determined according to the methods described in previous studies [59,60]. For proline determination, the extraction was performed using sulfosalicylic acid and then reacted with ninhydrin in a chromogenic reaction. The absorbance was measured at 520 nm, and the quantified proline was calculated as μg g^−1^. For 1-pyrroline measurement, the reaction solution contained 1,4-diaminobutane, and the optical density change was recorded at 430 nm. Methylglyoxal contents were determined by reacting the extracted solution with 5 M perchloric acid and 1,2-diaminobenzene (7.2 mM). The final values were expressed as mg g⁻^1^ after measuring the absorbance at 336 nm. The GABA contents were determined as described previously [61]. The 2-AP contents were measured as described in our previous study [60]. The ground plant samples were extracted with dichloromethane for 4 h. The supernatant was transferred to vials with a disposable micropipette and analyzed using a GCMS-QP2010 machine (Shimadzu Corporation, Kyoto, Japan) for GC-MS analysis.

### 2.8. Statical Analysis

Statistical analysis was conducted to identify significant differences between the control and treated plants and related indicators. The least significant difference (LSD) test was employed at a significance level of *p* < 0.05. All data are shown as means ± SD, derived from three replications for each treatment. Graphs were created using GraphPad Prism (Version 9.0).

## 3. Results

### 3.1. SA and MT Promote Photosynthesis and Growth-Related Attributes in Fragrant Rice Plants to Reduce Boron Toxicity

To evaluate the efficacy of SA and MT in lessening boron toxicity in fragrant rice seedlings, treatments with varying concentrations of SA and MT, both individually and in combination, were carried out. The concentrations tested included 0 µM control, 100 µM SA, 100 µM MT, and a combination of both. Boron toxicity levels were maintained at either 0 or 800 µM. Boron uptake in rice primarily occurs through root absorption and subsequent transport via transpiration streams, leading to accumulation in older shoots without significant translocation [62]. Consequently, the study parameters encompassed data from both roots and shoots of fragrant rice seedlings.

**Figure 1:**
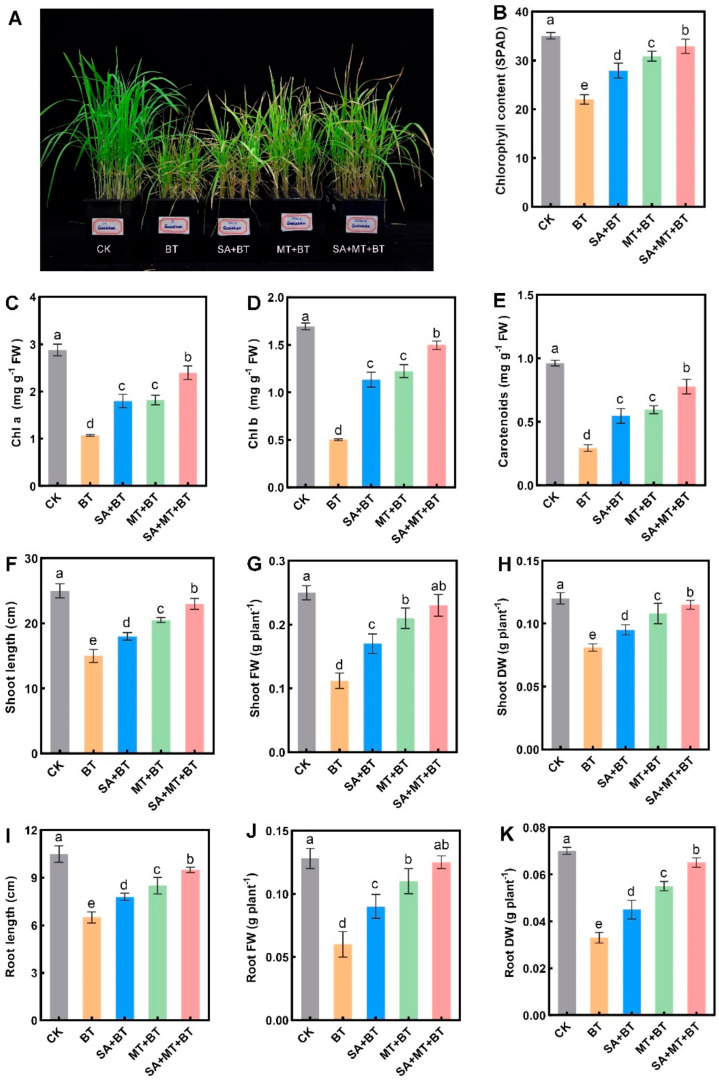
Impact of SA and MT on boron-induced alterations in rice seedling physiology and growth parameters. (**A**) Plant growth and treatment; (**B**–**E**) Changes in photosynthetic pigments; (**F**–**K**) Changes in plant biomass parameters such as length, fresh and dry weight. Data represents the mean of 3 replicates (n = 3), with vertical bars indicating the standard error (±SE). Dissimilar small alphabets on bar graphs denote significant differences between control and treatments (*p ≤* 0.05).

Fragrant rice plants exhibited stunted growth and chlorosis symptoms in response to boron toxicity (Figure 1A). Treatment with either SA or MT mitigated the stunted growth symptoms induced by boron toxicity. However, the co-application of SA and MT had a more pronounced protective effect compared to the individual treatments. Boron toxicity reduced the chlorophyll content SPAD index in rice plants by 38% compared to the control (Figure 1B). Nevertheless, foliar application of SA + boron, MT + boron, or the combination of both (MT + SA + boron) effectively alleviated boron-induced inhibition, resulting in increases in chlorophyll content SPAD index by 9.5%, 12.6%, and 15.3%, respectively, as compared to BT. Furthermore, the contents of Chl a, Chl b, and Carotenoids ranged between 70% and 120%, 110% and 150%, and 60% and 109%, respectively, when SA + boron, MT + boron, or the combination was applied. As expected, BT reduced Chl a, Chl b, and Carotenoids content by 60.58%, 75.42%, and 81.65%, respectively, when compared to the control group (Figure 1C–E).

Furthermore, boron toxicity notably diminished shoot length (SL), shoot fresh weight (SFW), and shoot dry weight (SDW), demonstrating significant reductions when compared to the control group (Figure 1F–H). Conversely, treatments with SA + boron, MT + boron, or MT + SA + boron led to significant increases in SL (12.3%, 20.3%, and 34.35%, respectively), SFW (21%, 33.7%, and 39.51%, respectively), and SDW (18.5%, 23.85%, and 26.23%, respectively), compared to only boron-treated plants.

Likewise, boron stress led to marked decreases in root length (RL), root fresh weight (RFW), and root dry weight (RDW) relative to the control group (Figure 1I–K). However, foliar application of SA + boron, MT + boron, or MT + SA + boron resulted in significant increases in RL (15%, 24.32%, and 35.7%, respectively), RFW (24.6%, 36.75%, and 47.51%, respectively), and RDW (18.5%, 34.85%, and 45.23%, respectively), compared to only boron-treated plants. Overall, our results indicate that both SA and MT treatments efficiently alleviate boron toxicity-induced effects on fragrant rice plant growth.

### 3.2. SA and MT Alleviate Boron Toxicity-Induced Defects in Chloroplast and Root Structures in Fragrant Rice

**Figure 2:**
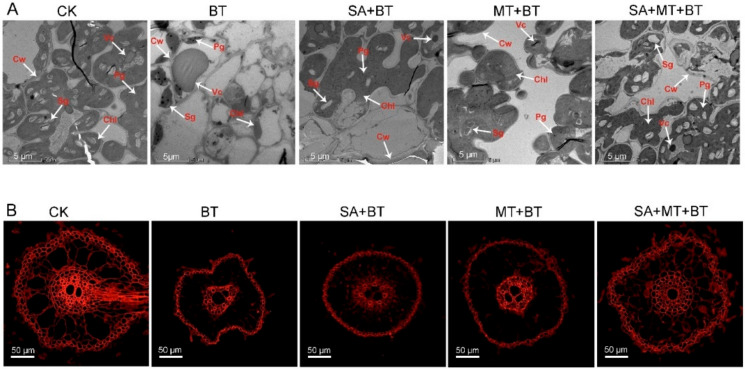
Impact of SA and MT on boron-induced changes in rice leaves chloroplast apparatus and root structure: (**A**) TEM and (**B**) confocal microscopic pictures of rice leaves and roots.

Our findings demonstrate that boron toxicity caused significant damage to the chloroplast integrity and ultrastructure configuration in fragrant rice plants. In response to boron toxicity, the chloroplasts exhibited a distorted structure, irregular shape, and reduced organization. These structural changes were accompanied by a noticeable reduction in starch grain accumulation, indicating impaired photosynthetic capacity and overall cellular health (Figure 2A). Nevertheless, chloroplast integrity and ultrastructure were significantly enhanced by treatments with SA, MT, and their mixture. Chloroplasts in SA + MT + boron-treated rice plants maintained a more consistent and regular oval shape, and the internal thylakoid membranes were better organized. Furthermore, in response to SA + MT + boron, there was a notable increase in the accumulation of starch grains within the chloroplasts (Figure 2A), highlighting the synergistic effects of SA and MT in mitigating boron toxicity-induced damage.

Furthermore, laser confocal microscopy analysis revealed severe damage to the root structure, including the root cortex cells and root cell walls, in response to boron toxicity relative to the control (Figure 2B). Boron toxicity led to disorganized root cortex cells and compromised root cell wall integrity, which are critical for nutrient uptake and overall root function. In contrast, the phenotype of roots treated with SA, MT, and SA + MT showed significant improvements. The root cells exhibited recovery of structural integrity, with well-organized cortex cells and intact cell walls. The SA + MT combination treatment was especially effective in enhancing root structure, further underscoring the synergistic benefits of these treatments in alleviating boron toxicity-induced root defects in root structure (Figure 2B).

Overall, our findings imply that SA, MT, and their combination can greatly lessen the harmful effects of boron toxicity on the root structures and chloroplasts of fragrant rice plants. The treatments not only restore cellular integrity and functionality but also promote overall plant growth and resilience under boron toxicity.

### 3.3. SA and MT Treatment Reduced Hydrogen Peroxide, Malondialdehyde, Electrolyte Leakage Percentage and Improved Anti-Oxidant Enzyme Activities under Boron Toxicity in Fragrant Rice

**Figure 3:**
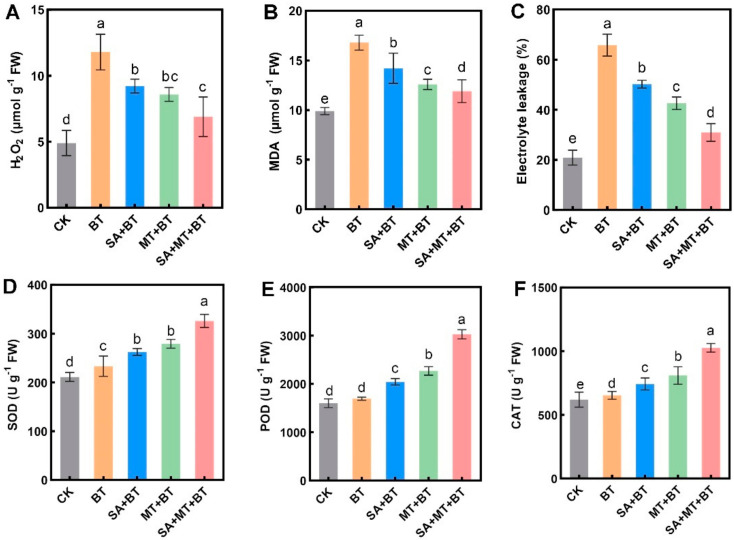
Impact of SA and MT on boron-induced changes in rice seedling physiological parameters: (**A**) H_2_O_2_ levels, (**B**) MDA levels, (**C**) electrolyte leakage, and (**D**–**F**) show antioxidant enzymes such as SOD, POD, and CAT. Data represent the mean of 3 replicates (n = 3) with vertical bars indicating ±SE. Dissimilar small alphabets on bar graphs denote significant differences between control and treatments (*p ≤* 0.05).

Under boron toxicity, levels of H_2_O_2_, MDA, and EL increased dramatically by 220%, 168%, and 324%, respectively, relative to control plants (Figure 3A–C). However, treatment with either MT or SA exhibited similar positive effects in alleviating these effects, bringing them closer to control levels. Specifically, SA treatment notably reduced H_2_O_2_, MDA, and EL levels by 36%, 40%, and 47%, respectively, relative to control plants (Figure 3A–C). Moreover, the combined application of SA and MT showed even greater efficacy, reducing H_2_O_2_, MDA, and EL by 100%, 90%, and 180%, respectively (Figure 3A–C). These findings suggest that while MT and SA each provide beneficial effects on rice plants under boron toxicity, the co-application of MT + SA synergistically amplifies the regulation of these parameters (Figure 3A–C).

Furthermore, we examined the effect of these treatments on the activity of key antioxidant enzymes, such as SOD, POD, and CAT, which serve as markers of oxidative stress. Interestingly, under boron toxicity, the activities of SOD, POD, and CAT remained relatively unchanged relative to the control (Figure 3D–F). The application of SA and MT independently elevated the activities of SOD (by 27–35%), POD (by 23–28%), and CAT (by 20–22%) relative to control plants. Remarkably, the combined treatment of SA and MT led to a more pronounced increase, enhancing the activities of SOD, POD, and CAT by 42%, 61%, and 83%, respectively, as compared to the control (Figure 3D–F). These outcomes underscore the potential of SA, MT, or their co-application to alleviate boron toxicity-induced stress in rice plants, with the co-treatment demonstrating enhanced effects on antioxidant enzyme activities (Figure 3D–F).

### 3.4. SA and MT Improve Ascorbate–Glutathione (AsA–GSH Cycle) Content, and Glyoxalase System under Boron Toxicity in Fragrant Rice

**Figure 4:**
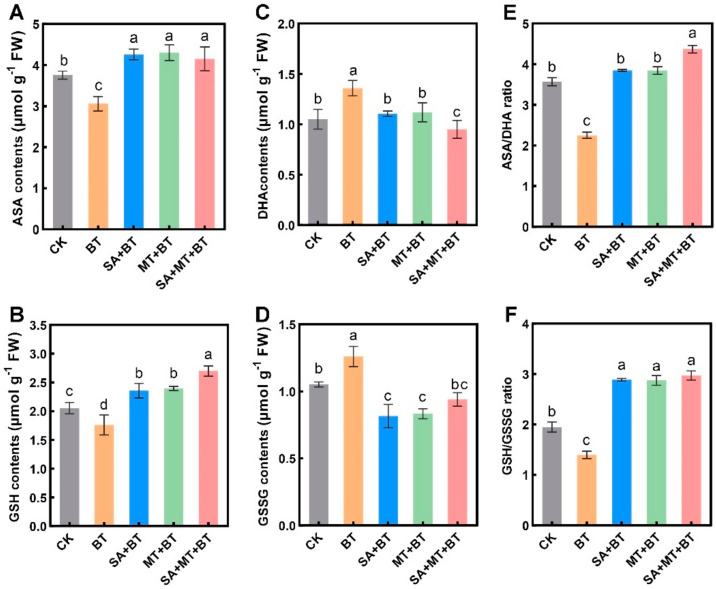
Impact of SA and MT on boron-induced changes in non-enzymatic antioxidants and oxidized glutathione in rice plants: (**A**) Ascorbate, (**B**) Dehydroascorbate (DHA), (**C**) Reduced glutathione (GSH), (**D**) Oxidized glutathione (GSSG), (**E**) Ratio of ASA/DHA and (**F**) Ratio of GSH/GSSG. Data represent the mean of 3 replicates (n = 3) with vertical bars indicating standard error (±SE). Dissimilar small alphabets on bar graphs denote significant differences between control and treatments (*p ≤* 0.05).

Under boron toxicity, the concentrations of Ascorbate and Glutathione in rice seedlings were significantly decreased by 27.25% and 17.34%, respectively (Figure 4A,B). This reduction in essential antioxidants highlights the detrimental impact of boron on the plant’s oxidative balance. However, treatments with SA and MT, either individually or in combination, showed promising effects in mitigating these reductions compared to control plants. SA treatment alone increased AsA and GSH levels by 18.25% and 19.28%, respectively, compared to the control treatment (Figure 4A–D), demonstrating SA’s effectiveness in enhancing antioxidant capacity under stress. Moreover, the combined application of SA and MT further enhanced these parameters, with GSH levels increasing significantly by 32.41% compared to the controls (Figure 4A,B).

Moreover, under boron toxicity, the concentrations of Dehydroascorbate and Oxidized Glutathione increased by 40.21% and 30.25%, respectively (Figure 4C,D). This increase in oxidized forms of antioxidants further underscores the oxidative damage induced by boron toxicity. However, SA and MT treatments, either individually or as a mixture, reduced the concentrations of DHA and GSSG by 13.23% to 20.14%. This reduction indicates an improved redox state in the treated plants. Additionally, in fragrant rice leaves, both under control conditions and boron stress, the provision of SA, MT, and their combination significantly elevated the ratio of AsA to DHA and GSH to GSSG (Figure 4E,F). These increased ratios indicate a more favorable redox balance, which is crucial for maintaining cellular health and function under stress conditions. These outcomes clearly demonstrate that SA and MT have the capability to improve the redox balance in plants affected by BT. The treatments enhance the levels of reduced antioxidants (AsA/GSH) while reducing the oxidized forms (DHA/GSSG), thereby contributing to a more robust antioxidant defense system. This improvement in redox homeostasis suggests a potential role for SA and MT in enhancing boron toxicity tolerance in fragrant rice plants, which could be crucial for developing strategies to mitigate the impacts of boron toxicity on crop productivity.

**Figure 5:**
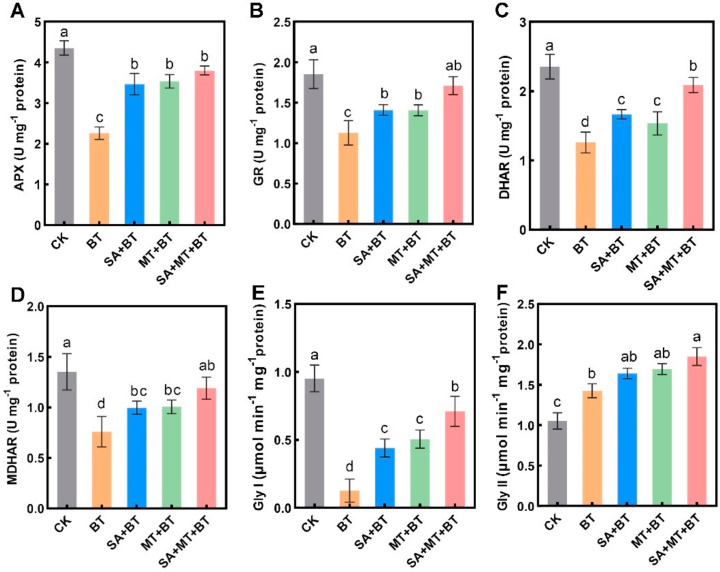
Impact of SA and MT on boron-induced changes in activities of enzymes involved in the Ascorbate–Glutathione (AsA–GSH) cycle and Glyoxalase system of rice plants: (**A**) Ascorbate Peroxidase (APX), (**B**) Glutathione Reductase (GR), (**C**) Dehydroascorbate Reductase (DHAR), (**D**) Monodehydroascorbate Reductase (MDHAR) and Glyoxalase I (Gly-I) (**E**) and Glyoxalase II (Gly-II) (**F**). Data represent the mean of 3 replicates (n = 3) with vertical bars indicating ±SE. Dissimilar small alphabets on bar graphs denote significant differences between control and treatments (*p ≤* 0.05).

Additionally, we examined the activity of pivotal enzymes APX, GR, DHAR, and MDHAR in the AsA–GSH pathway (Figure 5A–D). Boron toxicity led to a notable decrease in APX, GR, DHAR, and MDHAR activities by 56%, 45%, 51%, and 53%, respectively. This decline in enzymatic activity under boron toxicity underscores the oxidative stress and impaired antioxidant defense mechanism in fragrant rice plants. Nevertheless, treatment with SA and MT individually improved the activity of these enzymes. Specifically, the combined application of SA and MT resulted in a further augmentation of APX, GR, DHAR, and MDHAR activities by 43%, 34%, 41%, and 33%, respectively, compared to plants exposed to boron toxicity alone. Notably, treatment with SA, MT, or their mixture elevated the activity of all AsA–GSH cycle enzymes under boron toxicity. These conclusions highlight the vital role of upregulated AsA–GSH cycle enzymes induced by the joint application of SA and MT in improving plant tolerance to boron toxicity (Figure 5A–D).

Furthermore, under boron toxicity, Glyoxalase I (Gly-I) activity decreased substantially by 85%, while Glyoxalase II (Gly-II) activity presented a contrasting rise of 31% (Figure 5E,F). This indicates a reduction in Gly-I-mediated detoxification and a compensatory increase in Gly-II activity, highlighting an imbalance in the glyoxalase system under boron toxicity. Conversely, the application of SA, MT, or their combination effectively restored the Gly-I and Gly-II enzymes’ activities relevant to boron-treated plants. The MT + SA combination resulted in the most significant increases in Glyoxalase I (Gly-I) and Glyoxalase II (Gly-II) activities, showing respective increments of 275% and 26% compared to plants under boron toxicity. SA treatment led to increases of 175% and 12% in Gly-I and Gly-II activities, respectively, while MT treatment showed rises of 190% and 15% in Gly-I and Gly-II activities, respectively, compared to plants exposed to boron stress (Figure 5E,F).

These findings indicate that the collective application of SA and MT may synergistically improve rice plants’ tolerance to boron toxicity. The significant restoration and enhancement of Gly-I and Gly-II activities highlight the role of SA and MT in maintaining the balance and efficiency of the glyoxalase system under boron toxicity. The collective data from our study suggest that the upregulated activities of enzymes in both the AsA–GSH cycle and the glyoxalase system are induced by the joint application of SA and MT. These enzymatic enhancements lead to better management of oxidative stress and detoxification processes, contributing to enhanced growth and photosynthetic efficiency in boron-toxic rice plants.

### 3.5. SA and MT Treatment Restored 2-AP Biosynthesis under Boron Toxicity

**Figure 6:**
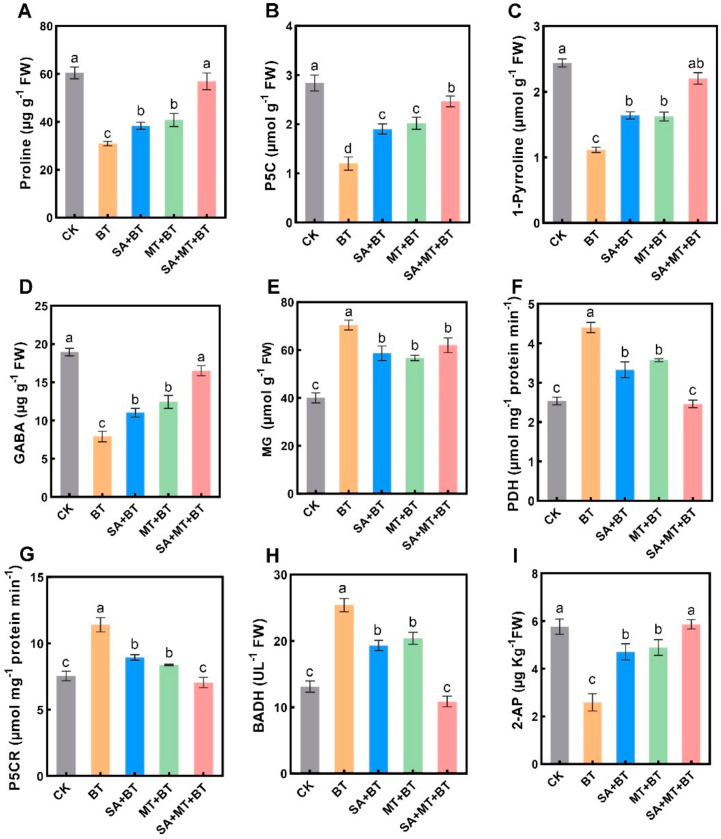
Impact of SA and MT on boron-induced changes in proline metabolism system in rice seedlings: (**A**) Proline content, (**B**) P5C content, (**C**) 1-pyrroline content, (**D**) GABA content and (**E**) methylglyoxal (MG). (**F**) PDH activity, (**G**) P5CR activity, (**H**) BADH activity, and (**I**) 2-AP content. Data represent the mean of 3 replicates (n = 3) with vertical bars indicating ±SE. Dissimilar small alphabets on bar graphs denote significant differences between control and treatments (*p ≤* 0.05).

We investigated the impact of boron toxicity on the scented traits of fragrant rice by examining its effects on the regulatory pathways involved in 2-AP biosynthesis, the compound responsible for the rice’s fragrance. To test this hypothesis, we quantified the levels of these precursors and measured the enzymatic activity of key enzymes involved during the 2-AP biosynthesis. The results displayed a substantial decline in proline, 1-pyrroline, GABA, and P5C contents by 50%, 53%, 73%, and 62%, respectively, under boron toxicity (Figure 6A–D), while there was a 45% rise in methylglyoxal (MG) content compared to control plants, indicating that boron toxicity adversely affects the aroma biosynthesis pathway of fragrant rice (Figure 6E). Remarkably, boron poisoning raised the activity of three enzymes implicated in this pathway: proline dehydrogenase (PDH), betaine aldehyde dehydrogenase (BADH), and pyrroline-5-carboxylate reductase (P5CR) by 45%, 38%, and 52%, respectively (Figure 6F–H). Despite this increase in enzyme activity, the reduction in precursor contents suggests a bottleneck at an earlier stage in the pathway or an increased degradation rate of these intermediates under boron toxicity. Finally, the quantification of 2-AP content in fragrant rice leaves confirmed that boron toxicity significantly reduced the 2-AP levels (Figure 6I). This decrease in 2-AP levels correlates with the reduced availability of its biosynthetic precursors, confirming that boron toxicity interferes with the aroma production in fragrant rice. Notably, applying SA, MT, and their combination (SA + MT) mitigated these negative effects. Treatments with SA and MT under boron toxicity resulted in notable increases in the levels of proline, P5C, 1-pyrroline, and GABA (Figure 3A–D). The combined application of SA and MT was particularly effective, leading to significant increases in proline (92%), P5C (110%), 1-pyrroline (105%), and GABA (90%) compared to boron treatment alone. Additionally, the combined treatment significantly modulated enzyme activities, reducing the over-activity of P5CR, PDH, and BADH by 40% to 150% compared to boron-stressed plants, thus normalizing the biosynthetic pathway (Figure 6F–H). As a result, 2-AP levels were successfully restored to those seen in control plants by applying SA and MT under conditions of boron toxicity (Figure 6I). This suggests that by raising proline concentration and perhaps encouraging its production, SA and MT are essential for reducing the deficiencies in fragrant rice’s scent caused by boron toxicity. These findings imply that the application of MT, SA, or their mixture can significantly enhance the aromatic properties of fragrant rice by alleviating the inhibitory effects of boron toxicity on 2-AP biosynthesis. However, more investigations are needed to completely clarify these processes and comprehend how SA and MT affect the synthesis of 2-AP in the presence of boron toxicity.

## 4. Discussion

As a vital micronutrient, boron is needed by plants for a variety of biochemical and metabolic processes [5]. However, when present in excessive amounts, boron toxicity can hinder crop development, leading to reduced yield potential. Previous research has highlighted the detrimental impact of boron toxicity on rice growth, resulting in decreased plant biomass [63]. In addressing this research gap, our research aimed to explore the synergistic actions of SA and MT treatments in enhancing growth, biomass production, and regulation of 2-AP biosynthesis under boron toxicity stress in fragrant rice. Prior studies have shown that individual applications of MT and SA can ameliorate the detrimental effects of abiotic stress on photosynthetic pigments in various crops [64,65,66]. Our study observed a decrease in chlorophyll contents in response to boron toxicity, and individual as well as combined application of SA and MT pointedly restored chlorophyll content. Furthermore, MT application has been shown in previous research to increase root and shoot weight and mitigate the detrimental effects of boron toxicity on rice plant growth, while SA has been shown to mitigate boron toxicity in wheat, thereby improving wheat growth [47,67]. Our current findings support these observations, demonstrating that plant growth and biomass were severely impaired under boron toxicity conditions. However, supplementation with SA/MT, and particularly with SA + MT in boron-stressed seedlings, mitigated the growth constraints. Notably, the joint treatment with SA and MT exhibited the most significant outcome on enhancing plant biomass, especially upper parts of rice seedlings, highlighting the cooperative potential of SA and MT in cultivating plant resilience to boron toxicity.

In response to unfavorable environmental conditions, plants often elevate the concentrations of ROS. Excessive ROS accumulation can trigger oxidative bursts or damage, disrupting numerous cellular processes [2,68]. To counteract this, plants rely on a defense mechanism composed of enzymatic/non-enzymatic mechanisms, which show a crucial role in scavenging reactive oxygen species [5]. Boron toxicity, for example, has been shown to elevate ROS concentrations, disrupting the mechanism of tedious electron transport in plant mitochondria [69]. However, a number of studies have shown that exogenous treatment with MT and SA can reduce oxidative stress caused by different circumstances [65,70]. Our study found that boron toxicity caused rice seedlings to have higher levels of H_2_O_2_, MDA, and EL, all of which are signs of oxidative stress. However, treatment with MT, SA, or their mixture exhibited promising results in reducing these markers of oxidative stress. These findings underscore the antioxidant properties of SA/MT and their potential as effective strategies for alleviating oxidative stress in plants subjected to boron toxicity. Plants have developed complex defensive systems against oxidative stress and control the build-up of ROS by employing enzymes such as SOD, APX, CAT, and POD [71]. MT and SA treatments have been recognized as potent antioxidants capable of scavenging oxygen free radicals [72]. This study corroborates these results by demonstrating that MT and SA treatments significantly enhance the antioxidant enzymes’ activities, thereby mitigating the detrimental effects of boron toxicity. Under boron stress conditions, the application of MT and SA led to a notable increase in the activity of antioxidant enzymes, as evidenced by our results. This aligns with recent research indicating that melatonin treatment protects safflower seedlings from lead toxicity. Similarly, by increasing antioxidant activity, salicylic acid protects watermelon plants against oxidative damage caused by boron [73,74]. The results of our investigation indicate that applying MT or SA helps plants under boron stress accumulate less ROS, as seen by the decrease in stress indicators. All things considered, the treatment of salicylic acid or melatonin may lessen the negative effects of boron toxicity by strengthening rice plants’ active oxygen scavenging system and boosting their resistance to stress. These findings demonstrate how MT and SA treatments may be used as useful tactics to reduce oxidative stress and enhance plant health when exposed to boron-induced damage.

The impact of boron toxicity on ascorbate and glutathione levels has been recognized, potentially leading to oxidative damage [75]. GSH and AsA are essential for initiating the antioxidant defense system since they are non-enzymatic antioxidants, enabling plants to acclimatize to environmental stresses [76,77]. These antioxidant molecules act as scavengers of ROS, thereby preserving cellular redox balance [78]. In our study, boron toxicity reduced the AsA and GSH (reduced forms) levels while increasing the levels of DHA and oxidized GSSH in fragrant rice plants. This oxidative impairment induced by boron toxicity may be linked with alterations in the redox potential of AsA and GSH, consistent with previous reports in potato plants under boron toxicity [79]. It is commonly known that AsA and GSH shield plants from oxidative stress [80,81]; GSH protects cell membranes from oxidative damage to lipids and proteins by activating glutathione-S-transferase [82]. However, similar to the effects observed in potatoes under boron toxicity [79] and wheat under drought stress [83], our results show that boron toxicity leads to an increase in DHA and GSSG levels, indicative of disturbances in cellular redox potential. Furthermore, the application of MT, SA, and MT + SA reversed the redox potential of AsA and GSH by elevating their contents and reducing DHA and GSSH levels under boron toxicity. This restoration of redox balance may contribute to ROS scavenging. Thus, the reduction in DHA and GSSH levels resulting from MT and SA treatment likely aids in ROS scavenging, thereby ameliorating the toxic impacts of oxidative stress in plants. These results coincide with earlier studies demonstrating that MT/SA treatments increase AsA and GSH contents while reducing the accumulation of oxidized forms of these antioxidants under boron toxicity conditions in wheat, maize, and oranges [84,85,86].

Under conditions of boron toxicity, fragrant rice plants showed a discernible decrease in the activity of AsA–GSH cycle enzymes, including APX, GR, DHAR, and MDHAR. These observations are parallel to the findings in wheat under boron toxicity [87]. Furthermore, the individual application of MT, SA, and their combination (MT + SA) demonstrated a reduction in boron accumulation in leaves through the modulation of the AsA–GSH cycle. An essential enzyme associated with the AsA–GSH cycle, APX, is mostly present in the stroma and membranes of plastids, where it uses AsA as an electron donor to change H_2_O_2_ into water [88]. The APX activities in fragrant rice leaves were reduced by boric acid toxicity, which is in line with findings in wheat [89], lettuce [90], and linseed [91]. However, conflicting findings suggest that boron toxicity may enhance APX enzyme activities in wheat [92] and rice [93]. Repressed APX activity can lead to surplus accumulation of H_2_O_2_ in different components of the cell, potentially damaging lipids and proteins [94]. Similar to rice under arsenic and boron stress, MT, SA, and their combination supplementation enhanced the APX activity in boron-stressed fragrant rice plants [47,93,95]. Since GSH acts as a scavenger of ROS in stressed plants, increasing tolerance to oxidative damage, GR activity is essential for restoring GSH levels and boosting cellular antioxidant capacity [96,97]. Similar to APX, boron toxicity decreased the GR activity in fragrant rice leaves, as observed in wheat [89], lettuce [90], and linseed [91]. In our experimental conditions, the decrease in GR activity induced by boron toxicity affected the ascorbate redox status, reducing stress tolerance. However, supplementation with MT, SA, and their combination led to an increased GR activity in boron-stressed fragrant rice plants, as previously observed in rice under arsenic and boron stress [47,93,95]. As demonstrated by *Trigonella foenum-graecum*, inconsistent findings have been noted, indicating that SA may lessen GR activity [98]. In the AsA–GSH pathway, two enzymes are involved: DHAR catalyzes DHA to AsA conversion, while MDHAR aids in maintaining the reduced pool of AsA and controls ascorbate’s redox state [99]. When citrus and fragrant rice leaves were exposed to boron stress, DHAR and MDHAR activities were decreased [100]. However, in wheat, divergent findings were noted, indicating that boron toxicity did not alter DHAR activity [89]. Supplementation with MT, SA, and their combination increased the activities of DHAR and MDHAR in boron-stressed fragrant rice plants, as observed in rice under arsenic and boron stress [47,93,95]. Furthermore, it has been shown that MT therapy increases enzymatic activity by triggering the expression of genes involved in MDHAR and DHAR production [101]. It is crucial to underline that under stressful circumstances, AsA concentrations and its redox status are crucially regulated by both DHAR and MDHAR [102].

Gly-I and Gly-II are two essential enzymes that are part of the Glyoxalase system and work together to prevent plants from accumulating too much methylglyoxal (MG) when they are stressed [103,104]. Similar to findings in rice under arsenic stress, MG accumulation in fragrant rice leaves was exacerbated by boron poisoning [105]. Increasing the activity of enzymes associated with the glyoxalase system can improve stress tolerance in plants [106,107]. In this study, boron toxicity led to elevated levels of methylglyoxal and Gly-II activity while decreasing Gly-I activity. Despite the increase in Gly-II activity in boron-treated plants, it was insufficient to detoxify MG, aligning with findings from previous studies in wheat and rice under abiotic stress conditions [108,109]. The MT/SA application markedly boosted enzyme activities while decreasing MG levels, indicating effective MG detoxification and enhanced tolerance to boron toxicity. The findings align with earlier research that indicates MT can augment Gly-I and Gly-II activity in potato plants experiencing water stress [110]. Moreover, it has been demonstrated that applying MT to heat-stressed maize plants reduces the formation of MG via increasing glyoxalase system activity [111]. Furthermore, it has been proven that SA increases the glyoxalase system, which enhances the resistance of soybean plants to waterlogging stress [112]. The heightened activity of Gly-I and II observed in fragrant rice plants exposed to boron toxicity and supplemented with MT and SA could be attributed to the enhanced stimulation of GSH production by these signaling molecules.

Proline serves as a crucial osmolyte, which is accumulated during environmental stress to maintain osmotic pressure homeostasis. Proline is also a crucial precursor of 2-AP, and 2-AP synthesis in fragrant rice is strongly correlated with proline metabolism [113]. MT is renowned for its ability to bolster the activities of enzymes pivotal in photosynthesis and carbon metabolism, alongside its role in amplifying the activity of P5CR, an enzyme pivotal in proline synthesis. Similarly, SA acts as a pivotal signaling molecule, contributing significantly to the mitigation of metalloid stress [95]. Our findings show that proline and 2-AP levels under boron toxicity were positively correlated with the application of MT, SA, and their combination (SA + MT). The stimulation of the proline biosynthesis pathway is probably the cause of this increase in proline concentration. P5C is an intermediate precursor in both proline biosynthesis and degradation [114]. MT and SA treatments likely improve plant growth under boron stress by enhancing proline synthesis, achieved through the suppression of PDH and P5CR activity. This is consistent with previous findings indicating that MT enhances proline levels in rice seedlings under boron toxicity [47], while SA applications induce proline accumulation to mitigate the effects of boron toxicity by scavenging ROS and activating antioxidant defense systems [85]. Additionally, compared to separate treatments, the combination of MT and SA showed synergism on these parameters, indicating possible crosstalk between SA and MT in controlling plants’ response to boron stress. Previous research has highlighted the way in which MT and SA interact with other signaling molecules, demonstrating how these interactions affect stress tolerance and plant development. For example, it has been demonstrated that MT increases SA production and signaling, suggesting a positive feedback loop between the molecules [115]. In the present study, we deduced that the inhibition of proline degradation under low SA and MT levels induced negative feedback regulation in proline biosynthesis, causing the accumulation of P5C. This suggested more P5C could be involved in 2-AP biosynthesis, thus increasing the 2-AP level in scented rice.

**Figure 7:**
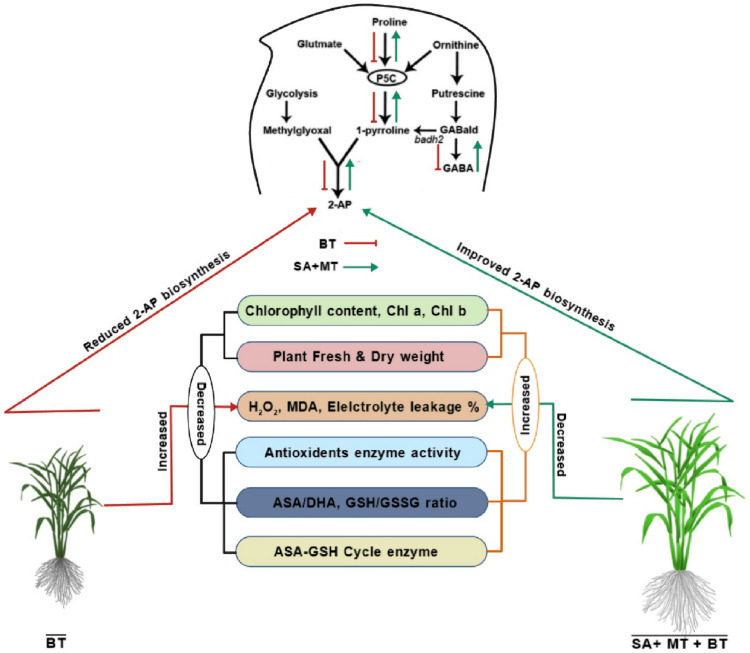
Schematic illustration of salicylic acid and melatonin-mediated 2-AP biosynthesis under boron toxicity in fragrant rice.

Moreover, the observed response of 2-AP content to boron toxicity in aromatic rice might be due to alterations in BADH activity [116,117]. In our study, we found a significant negative relationship between 2-AP content and BADH activity, which aligns with previous research [118]. This inverse relationship suggests that higher BADH activity under boron toxicity may lead to lower 2-AP production. Moreover, the content of GABA, a precursor in the 2-AP biosynthesis pathway that can be formed by BADH, decreased significantly under boron toxicity and showed a positive correlation with 2-AP content [119]. This finding is consistent with Mo et al.’s [61] study but contrasts with Bao et al.’s results [59], indicating variability in the response of GABA and 2-AP under different stress conditions or experimental setups. We deduced that SA and MT might enhance GABA biosynthesis through alternative pathways, leading to a negative feedback regulation that down-regulates BADH activity (Figure 7). This down-regulation could, in turn, enhance 2-AP biosynthesis (Figure 7). The increase in proline, P5C, 1-pyrroline, and GABA levels upon SA and MT treatment supports this hypothesis, suggesting that these compounds can modulate multiple aspects of the 2-AP biosynthesis pathway beyond just influencing BADH activity.

## 5. Conclusions

Our findings demonstrate that the combined application of SA and MT can effectively mitigate the detrimental effects of boron toxicity on fragrant rice by modulating multiple biochemical pathways. The improvements in the AsA–GSH cycle and glyoxalase system, along with the enhancement of 2-AP biosynthesis, underscore the potential of these treatments in promoting stress tolerance and maintaining desirable traits, such as aroma, in fragrant rice. Conclusively, melatonin and salicylic acid synergistically alleviate boron toxicity-induced disruptions on the 2-AP biosynthesis pathway by improving the 2-AP precursors and enzymatic activities, as well as modulating the physio-biochemical processes and antioxidant defense system of fragrant rice plants. Further research in field conditions might validate the synergistic effects of MT and SA on the 2-AP contents in grains and other grain quality traits of fragrant rice.

## Data Availability

The original contributions presented in the study are included in the article, further inquiries can be directed to the corresponding author/s.
